# Applying Genomic Epidemiology to Characterize a COVID-19 Outbreak in a Developmentally Disabled Adult Group Home Setting, Arizona

**DOI:** 10.3389/fpubh.2021.668214

**Published:** 2021-05-13

**Authors:** Hayley D. Yaglom, Marette Gebhardt, Ashlyn Pfeiffer, Mary Ellen Ormsby, Daniel E. Jasso-Selles, Darrin Lemmer, Megan L. Folkerts, Chris French, Matthew Maurer, Jolene R. Bowers, David M. Engelthaler

**Affiliations:** ^1^Pathogen and Microbiome Division, Translational Genomics Research Institute, Flagstaff, AZ, United States; ^2^Coconino County Health and Human Services, Flagstaff, AZ, United States

**Keywords:** genomic epidemiology, developmental disabilities, public health, COVID-19, outbreak

## Abstract

Individuals living in congregate settings, including those in group homes, have been disproportionately impacted by COVID-19 and may be at increased risk of exposure or infection due to underlying illness. In mid-May 2020, local public health officials responded to an outbreak of COVID-19 among staff and residents associated with a multi-residential group home that provides care for adults with intellectual and developmental disabilities. Samples were collected at 16 of the homes. In four of the homes all the residents tested positive, and in the remaining 12 houses where samples were collected, all residents tested negative. Of the 152 individuals tested, 15/58 (25.9%) residents and 27/94 (28.7%) staff were positive for SARS-CoV-2, including eight hospitalizations and four deaths. Phylogenetic analysis of genomes from this outbreak in the context of genomes from Northern Arizona shows that very few mutations separate the samples from this outbreak. A potential transmission network was developed to illustrate person-place epidemiologic linkages and further demonstrates the dynamic connections between staff and residents with respect to each group home location. Epidemiologic and genomic evidence correlate, and suggest that asymptomatic infected staff likely introduced and spread COVID-19 in this setting. Implementation of public health prevention measures alongside rapid genomic analysis can help guide policy development and guide management efforts to prevent and mitigate future outbreaks.

## Introduction

The COVID-19 pandemic has dramatically impacted individuals in many different congregate settings, including long-term care facilities, homeless shelters, and group homes. Adults with intellectual or developmental disabilities (IDD) are three times more likely to suffer from underlying medical conditions, such as heart disease, diabetes, and respiratory illnesses, that are known COVID-19 risk factors, than those without IDD (1–4). It is also typical for people with IDD to have multiple chronic health conditions, which paired with metabolic and nutritional disorders, elevate the risk of experiencing more severe outcomes of COVID-19. Another analysis showed that COVID-19 patients with IDD, regardless of age, had the highest likelihood of dying from the virus (5). As of June 2020, it is estimated that more than 7,000 IDD congregate- setting residents have been diagnosed with COVID-19 nationwide, with at least 700 deaths (6). Furthermore, New York health officials have reported infection rates in group homes to be five times higher than the general population (7). Despite these numbers, which are likely an underestimation of the true burden on this population, limited scientific reports have highlighted outbreaks in group homes throughout the United States that care for individuals with IDD.

Arizona has reported COVID-19 cases associated with over 2,000 congregate settings. These cases represent a disproportion of the more than 550,000 cases statewide documented between January and December 2020 (8). While it is recognized that these populations have also experienced disproportionate morbidity and mortality rates, limited reports specifically describe outcomes experienced by individuals with IDD (4, 9, 10). Here, we describe an epidemiologic investigation paired with genomic analysis of a COVID-19 outbreak associated with multiple group home residences in Arizona.

## Methods

### Public Health Investigation

On May 15th 2020, public health officials were notified of positive COVID-19 cases associated with a multi-residence group home that provides services for people with IDD. In response, enhanced testing was conducted on May 26th and 27th in resident homes and at an on-site event. This organization has 21 locations throughout Northern Arizona. Each unit houses 2–6 residents that have their own bedrooms, and spend varying amounts of time in shared common areas. Each home is supported by 2–6 medical assistants and caregivers, some of whom work at multiple homes.

Sample collections and testing of residents and staff in the early weeks of the outbreak were performed at healthcare facilities and *via* a community collection site. Extensive contact tracing and collaboration with other public health agencies allowed for identification of all individuals linked with this outbreak. In total, 152 nasopharyngeal swabs collected from 58 residents living in 16 homes and 94 staff were submitted for SARS-CoV-2 PCR testing. Collection dates ranged from April 24 to June 11. Sampling did not occur at five additional locations managed by this organization, as they are used for recreational activities only or are located in another region of Northern Arizona and were not a part of this outbreak.

Public health, working closely with the facility management, collected information on clinical signs, timeline of the outbreak, and exposures of residents and staff working in the homes. The index resident cases in Houses A through D were identified on May 14, 15, 21, and 22, respectively. These houses are located within four to seven miles of each other, experienced a 100% residential infection rate (e.g., all residents in these houses tested positive), and were deemed “positive” houses. Positive resident and staff case samples were identified first in Houses A and B (5/7-5/27), followed by Houses C and D (5/15-6/2). The remaining 12 houses at which samples were collected were classified as “negative” houses (all residents tested negative, although some staff working in these homes (E–G) were positive with collection dates ranging the span of the outbreak, 4/24-6/1). House B initially had two residents; both tested positive and one suffered a severe clinical outcome resulting in death. The surviving resident was transferred to a different home that already had positive residents. None of the other residents were moved between homes throughout this outbreak.

A majority of the staff initially interacted with residents from multiple homes; however, upon identification of additional cases in Houses A and B, staff were assigned to work exclusively at one home. Seven staff that provided care for COVID-19 positive residents were provided alternative housing to avoid exposing their families and close contacts. Strategies to manage COVID-19 in group homes, as well as guidance on isolation, mask efficacy, quarantine, and enhanced personal protective equipment use were provided to the facility on May 22. Daily temperature checks, self-screening for staff, and comprehensive infection prevention procedures were employed to contain the spread once identified in these homes.

### Genomic Sequencing and Analysis

RNA was extracted using previously described methods (11, 12) and prepared for whole genome sequencing. SARS-CoV2 cDNA was amplified following the nCoV-2019 sequencing protocol V.124 and using the ARTIC v3 primer set, prepared for sequencing with plexWell384 (SeqWell), and sequenced on a NextSeq 550 with v2 chemistry and 150 X 150 base-pair reads (Illumina). Data were processed and virus genome consensus sequences were built using the Amplicon Sequencing Analysis Pipeline (ASAP) (12). Maximum likelihood phylogenetic trees containing the outbreak genomes and a subset of other Arizona genomes for context were generated using the Wuhan-1 genome as a reference using NextStrain (13, 14). The subset of genomes used was chosen using genome-sampler (15), which selects the most closely related samples from an available dataset collected within the same geographic region and time period.

### Epidemiologic Network

Staff and resident cases were loaded into MicrobeTrace (16) as a “Node List” and connections to their respective facilities were loaded as a “Link List” in comma-separated formats. Once loaded in MicrobeTrace: (1) node shapes were mapped to a column distinguishing between persons and places, (2) node labels were mapped to a column populated with a deidentified location ID for all locations, while this column remains empty for all nodes representing persons, (3) node colors were mapped to a column describing the patient outcome, (4) the timeline feature was controlled using the sample collection date as input from the “Node List” file, and finally (5) the graphic was exported as SVG objects at each time interval of interest. The SVG objects exported from MicrobeTrace were further augmented in Inkscape with an additional visualization layer to flag the most interconnected asymptomatic individuals in the network and to customize the figure's legend.

## Results

Of the 58 residents sampled, 15 (25.9%) tested positive. Residents ranged in age from 35 to 71 years (mean age = 56 years). None of the residents experienced the hallmark signs of COVID-19 (e.g., fever, cough, shortness of breath); however, staff reported that several infected residents were hypoxic and lethargic. Nine infected residents were confirmed to be asymptomatic at the time of sample collection. Among those who tested positive, 6/15 (40%) were hospitalized, and 4/15 (26.7%) died. The four residents that died ranged in age from 57 to 71 years old and all were reported to be immunocompromised and had extensive co-morbidities prior to becoming infected with COVID-19. These co-morbidities included, but were not limited to, hypothyroidism, seizure disorders, asthma, and previous diagnosis of cancer and tuberculosis in two of the four residents. Twenty-seven of ninety-four staff tested positive (28.7%); two were hospitalized and the remaining were either asymptomatic or developed only mild symptoms (Table 1).

**Table 1:** Demographic characteristics of COVID-19 positive staff and residents linked to a developmentally disabled adult group home setting.

	**Resident (*n* = 15) No. Positive (%)**	**Staff (*n* = 27) No. Positive^**+**^ (%)**
**Age (years)**		
<25	0 (0)	4 (14.8)
25–34	0 (0)	13 (48.2)
35–44	2 (13.3)	4 (14.8)
45–54	4 (26.7	3 (11.1)
55–64	6 (40.0)	2 (7.4)
65+	3 (20.0)	1 (3.7)
**Sex**		
Female	7 (46.7)	22 (81.5)
Male	8 (53.3)	5 (18.5)
**Associated Home**		
House A	4 (26.7	11 (40.7)
House B	2 (13.3)	6 (22.2)
House C	6 (40.0)	9 (33.3)
House D	3 (20.0)	4 (14.8)
House E	0 (0)	6 (22.2)
House F	0 (0)	5 (18.5)
House G	0 (0)	6 (22.2)
**Outcome**		
Asymptomatic	8 (53.3)	12 (44.4)
Hospitalized	6 (40.0)	2 (7.4)
Death	4 (26.7)	0 (0)

*+Number of positive staff associated with individual houses were not mutually exclusive. Staff were often assigned to work in more than one “positive” home*.

**Figure 1:**
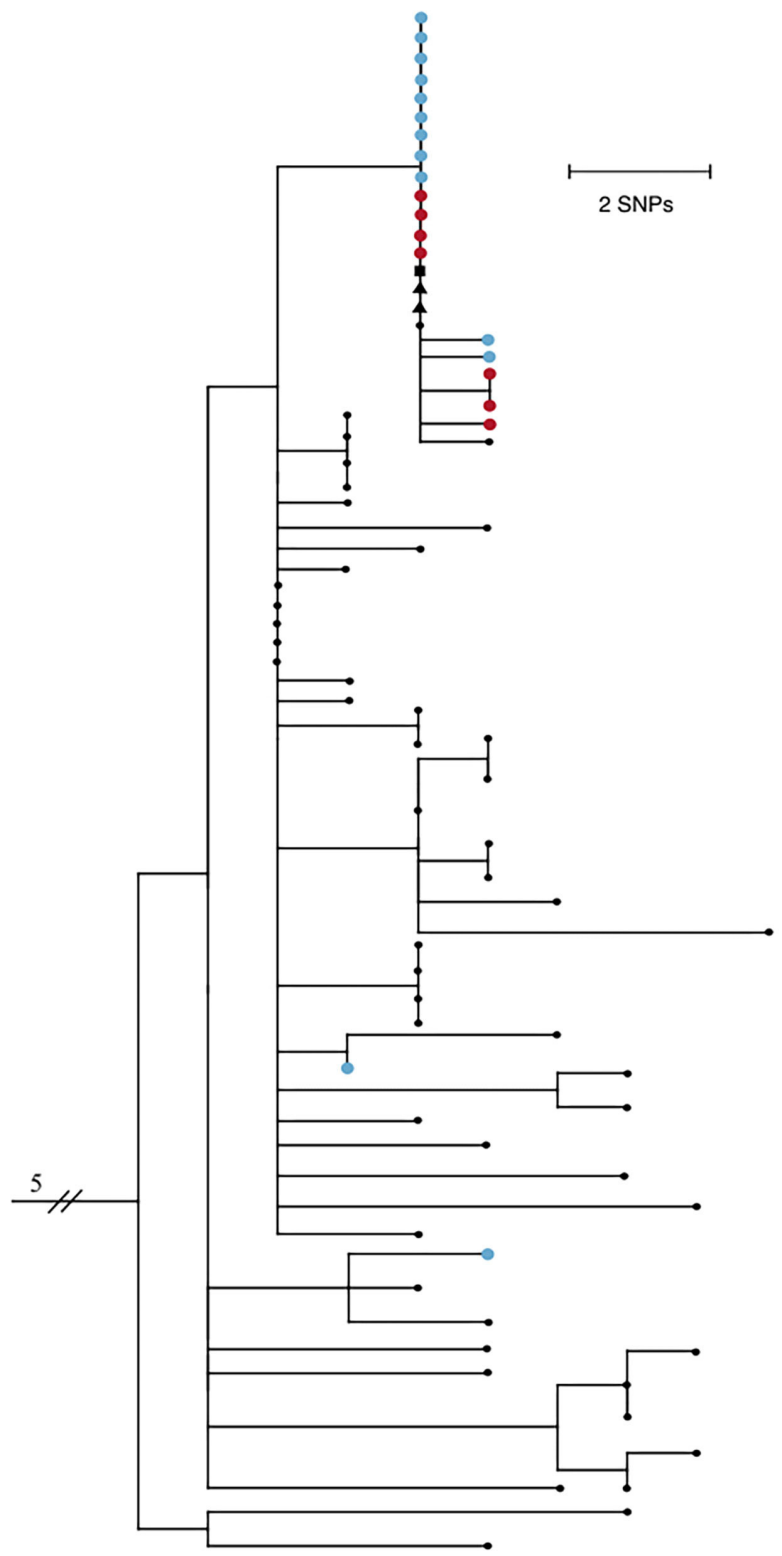
Maximum likelihood phylogenetic tree of 74 SARS-CoV-2 genomes from Northern Arizona, May-June 2020 generated by Nextstrain (13, 14) using the Wuhan1 genome as a reference (EPI_ISL_402125), showing 18/20 samples sequenced from this outbreak form one tight clade. Blue nodes represent sequences from staff and red nodes represent sequences from residents. Square shaped nodes represent the household contact of an infected staff and triangular shaped nodes represent healthcare workers. Genomes have been published to GISAID. EPI_ISL_694009-023, 025–040, 228, 231–235, 237–239, 241–242, 244, 318, 320, 322, 324, 328, 330, 335.341, 342, 345, 350, 351, 355, 378, 380, 381, 387, 389, 391, 398, 399, 434, 437, 442, 451, 455, 601, 607, 914212, 299. All Arizona samples in the tree have the D614G mutation.

Twenty-five of the forty-two positive samples (59.5%) were available for viral genome sequencing with Ct values ranging from 18.0 to 37.5. There was no observable difference in viral load between symptomatic and asymptomatic individuals. 20/25 samples had 90% or greater breadth of coverage across the SARS-CoV-2 genome at ≥10X depth of coverage. Phylogenetic analysis based on single nucleotide polymorphisms (SNPs) comprised a subset of Arizona SARS-CoV-2 genomes, and included five that were previously shown to cluster with the outbreak group. Results show the majority of genomes associated with this outbreak fall into a monophyletic clade defined by 2 distinct SNPs, C13860T and C21575T, the latter of which confers an L5F amino acid substitution in the spike protein gene (Figure 1).

Virus genomes from two staff exposed outside the workplace are not closely related to the others, indicating they are not part of the transmission network of this outbreak and did not seed the outbreak, while five community samples are clonal to this group, showing that this outbreak was not confined solely to the group home. Two of the five samples are from healthcare workers, one is a confirmed household contact of a staff member, and two additional samples have no known epidemiological connections. Residents did not have outside interactions other than receiving necessary medical care, including at the same healthcare facility where the above two healthcare workers were employed. Collection dates of the earliest staff cases precede both the resident and community cases; therefore, this transmission network was likely fueled by staff encounters in the group homes and community. Ongoing viral sequencing efforts of positive samples in subsequent months in the region revealed no additional cases associated with this outbreak.

**Figure 2:**
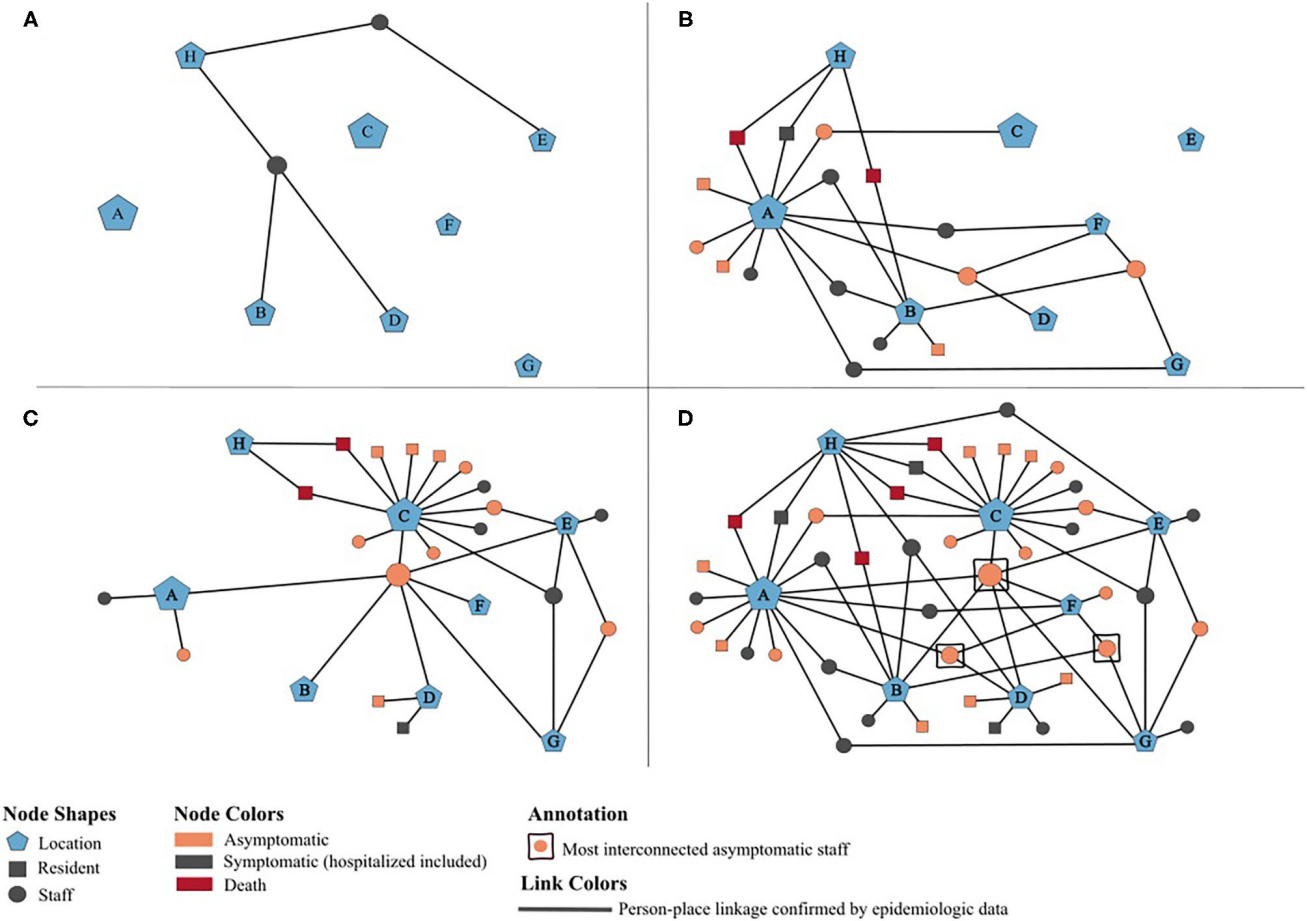
Potential transmission network of 42 SARS-CoV-2 positives cases associated with an outbreak at an adult group home setting, May-June 2020. **(A–D)** illustrate person-place linkages of staff and residents throughout the weeks of the outbreak. **(A)** Two initial positive staff pre-outbreak, May 1–10. **(B)** Staff associated with additional homes and residents in houses A and B test positive, May 11–20. Public health notified on May 15. **(C)** Residents in houses C and D test positive, May 21–30. Enhanced testing by public health on May 26–27. **(D)** Complete network of all positive cases at the end of the outbreak and associated houses, May 31–June 30.

To further characterize and understand the dynamics of this outbreak, a potential transmission network was developed using MicrobeTrace (16), which incorporates person-place linkages of all 42 positive cases ascertained through public health investigations and contact tracing (Figure 2). The timeline of the network relies on the earliest collection dates for the positive case samples. Each node is sized according to the number of person-place connections (e.g., more cases are associated with House A than House B). The letters reference the residential homes (A–G) and the hospital (H). Panels A–C illustrate the positive individuals and their associated locations by week of the outbreak, with panel D demonstrating the complete network highlighting the interactions and connections between staff with respect to each group home location and its residents. The network also displays the hypothesized movement of the virus throughout the homes, and indicates that asymptomatic staff connected to multiple homes likely played a significant role in sourcing this outbreak.

## Discussion

People with IDD often require a high level of direct care, may be unable to communicate symptoms of illness, and are dependent on close physical contact with support staff; thus, coping with the COVID-19 pandemic has been especially challenging for this demographic. Social distancing in this setting is not always feasible; therefore, despite measures taken to protect their patients and limit potential spread, caregivers can pose risk to residents. In this case study, we highlight an outbreak involving 42 individuals linked by a multi-residential group home environment that cares for adults with IDD.

While many staff were not in frequent close contact with one another in the work setting, several were housemates. Additionally, at least six were exposed through other means (e.g., family gatherings). Several staff also had close connections with Native American communities experiencing high COVID-19 attack rates during this timeframe. Given the continual risk of exposure both in and outside of the workplace and common practice for staff to work in multiple houses, it was difficult for public health officials to determine the most appropriate timeframe for quarantining and testing of staff. Furthermore, since many of the early cases were asymptomatic, our understanding of the variation of viral spread before and after implementation of distancing, isolation, and prevention measures relies on dates of collection (as mentioned above for the network) versus dates of symptom onset for positive case samples.

Sequencing data were not available for every positive case, a well-understood limitation when conducting genomic epidemiologic analyses, making it difficult to infer informative transmission maps. However, while a clear transmission pattern could not necessarily be ascertained through the genomics alone, the phylogeny of the outbreak shows a highly connected genomic network, and heightens the importance of using epidemiologic information in the context of the sequencing data when interpreting findings. Furthermore, public health was able to gather evidence. Overall, genomic and epidemiologic evidence supports our hypothesis and suggests that infected staff introduced COVID-19 into this setting, played a role in spreading the virus among the multiple homes, and contributed to limited community transmission.

Despite these challenges, enhanced precautions required of staff and timely interventions by the facility and public health curbed this outbreak. After the implementation of these measures on May 22 and widespread testing on May 26–27, only a small number of individuals tested positive (6/42; 14%). Given the vulnerable nature of people living in congregate settings, it is critical to have policies and procedures in place to manage disease outbreaks. Since the May outbreak, a number of prevention measures continue to be implemented by the organization, and are proving to be successful at mitigating the spark of new clusters or outbreaks, as there has only been a few sporadic cases in staff members. These measures specifically include oxygen and temperature checks on every resident multiple times throughout the day, daily temperature checks on staff, enhanced monitoring of staff exposures outside of work followed by at home isolation, limitation of visitors, and thorough cleaning of homes. Staff are now assigned to working at no more than two houses, and any staff that work at a higher risk home only provide care for residents in that home. Ongoing widespread screening of staff and residents is also occurring in partnership with public health to ensure early identification of potential asymptomatic infected individuals. Early interventions, paired with rapid genomic epidemiologic analyses, can provide a better understanding of transmission patterns and further guide public health efforts.

## Data Availability Statement

The genomic data supporting the conclusions of this article are publicly available from GISAID. Additional data will be made available by the authors without undue reservation, upon request.

## Ethics Statement

Ethical approval for this study and written informed consent from the participants of the study were not required in accordance with local legislation and national guidelines. This work was conducted in collaboration with a local public health agency as a public health surveillance activity, involving genomic sequencing and analysis of de-identified remnant biospecimens, and therefore is exempt from needing human subjects research board approval.

## Author Contributions

HY coordinated the work between the two agencies and prepared the manuscript alongside JB. AP and DJ-S assisted with development of the figures. MG, MO, and MM conducted the investigations and provided epidemiologic data. AP, DJ-S, MF, and DL performed genomic sequencing and data analysis. JB and DE oversaw the genomic response efforts and provided critical revisions of the manuscript for important intellectual content. All authors contributed to the article and approved the submitted version.

## Conflict of Interest

The authors declare that the research was conducted in the absence of any commercial or financial relationships that could be construed as a potential conflict of interest.
